# Chronic Mild Stress and Venlafaxine Treatment Were Associated with Altered Expression Level and Methylation Status of New Candidate Inflammatory Genes in PBMCs and Brain Structures of Wistar Rats

**DOI:** 10.3390/genes12050667

**Published:** 2021-04-29

**Authors:** Katarzyna Bialek, Piotr Czarny, Paulina Wigner, Ewelina Synowiec, Gabriela Barszczewska, Michal Bijak, Janusz Szemraj, Monika Niemczyk, Katarzyna Tota-Glowczyk, Mariusz Papp, Tomasz Sliwinski

**Affiliations:** 1 Laboratory of Medical Genetics, Faculty of Biology and Environmental Protection, University of Lodz, 90-236 Lodz, Poland; biaalek.k@gmail.com (K.B.); ewelina.synowiec@biol.uni.lodz.pl (E.S.); gabriela.barszczewska@edu.uni.lodz.pl (G.B.); 2 Department of Medical Biochemistry, Medical University of Lodz, 92-216 Lodz, Poland; piotr.czarny@umed.lodz.pl (P.C.); janusz.szemraj@umed.lodz.pl (J.S.); 3 Department of General Biochemistry, Faculty of Biology and Environmental Protection, University of Lodz, 90-236 Lodz, Poland; paulina.wigner@biol.uni.lodz.pl; 4 Biohazard Prevention Centre, Faculty of Biology and Environmental Protection, University of Lodz, 90-236 Lodz, Poland; michal.bijak@biol.uni.lodz.pl; 5 Institute of Pharmacology, Polish Academy of Sciences, 31-343 Krakow, Poland; niemczyk@if-pan.krakow.pl (M.N.); ktech@o2.pl (K.T.-G.); nfpapp@cyfronet.pl (M.P.)

**Keywords:** depression, chronic mild stress, venlafaxine, inflammation, expression, methylation

## Abstract

Preclinical studies conducted to date suggest that depression could be elicited by the elevated expression of proinflammatory molecules: these play a key role in the mediation of neurochemical, neuroendocrine and behavioral changes. Thus, this study investigates the effect of chronic mild stress (CMS) and administration of venlafaxine (SSRI) on the expression and methylation status of new target inflammatory genes: TGFA, TGFB, IRF1, PTGS2 and IKBKB, in peripheral blood mononuclear cells (PMBCs) and in selected brain structures of rats. Adult male Wistar rats were subjected to the CMS and further divided into matched subgroups to receive vehicle or venlafaxine. TaqMan gene expression assay and methylation-sensitive high-resolution melting (MS-HRM) were used to evaluate the expression of the genes and the methylation status of their promoters, respectively. Our results indicate that both CMS and chronic treatment with venlafaxine were associated with changes in expression of the studied genes and their promoter methylation status in PMBCs and the brain. Moreover, the effect of antidepressant administration clearly differed between brain structures. Summarizing, our results confirm at least a partial association between TGFA, TGFB, IRF1, PTGS2 and IKBKB and depressive disorders.

## 1. Introduction

Being one of the most frequently diagnosed mental diseases, depression (Major depressive disorder, MDD) affects more than 260 million people worldwide and is a significant contributor to the global burden of disease. Due to the constantly increasing number of patients, MDD is estimated to be the second leading cause of social disability. Depression reduces people’s functioning by inducing persistent sadness, lack of interest and anxiety. These and other symptoms often become chronic or recurrent and may lead to suicide [1]. Furthermore, above one-third of patients do not respond to antidepressant treatment [2,3].

Despite its importance, the pathogenesis of depression is not fully understood. Nevertheless, there is a growing body of evidence suggesting that it may be influenced by the activation of the immune system. One mechanism that has been proposed for its development is given in the “cytokine hypothesis” [4]; briefly, MDD could be elicited by the elevated expression and activity of proinflammatory molecules: these act as neuromodulators and thus play a key role in the mediation of neurochemical, neuroendocrine and behavioral changes [5]. Indeed, patients affected with medical conditions associated with chronic inflammation, i.e., rheumatoid arthritis, cardiovascular diseases and autoimmune disorders, are at higher risk of depression [6]. Moreover, a great amount of evidence confirms a link between inflammation and depression in patients without other medical conditions. A rich body of research indicates that MDD patients exhibit increased concentrations of cytokines and other proinflammatory markers, such as acute phase reactants, chemokines and adhesion molecules [7,8,9,10].

Patients with depression have also demonstrated activation of microglia, i.e., immune cells resident within the central nervous system (CNS) [11]. This may also contribute to neuroinflammation, and neurotrophic system disruptions since activated microglia express proinflammatory cytokines [12]. Additionally, their mobilization is connected with the activation of nuclear factor-kB (NF-kB), which is often responsible for cytokine production [13]. However, sometimes microglia exert a neuroprotective effect by releasing anti-inflammatory molecules, including transforming growth factor β (TGFB), to antagonize inflammation-promoted CNS damage [12].

TGFB is a class of polypeptide growth factors, which together with transforming growth factor α (TGFA), constitute the TGF family. Their main functions are embryonic development and regulation of immune system reactions [14,15]. TGFB is known to play a role in brain inflammation, as well as in the peripheral immune response [16,17]. In addition, TGFB can exert neuroprotective effects in many neurodegenerative disorders [18]. However, reports about its role in MDD are inconsistent. Its level has been found to be increased in animal studies, with this increase being associated with an imbalance between Treg and Th17 cells [19], while other studies have identified lower TGFB expression in depressed patients than in healthy subjects [20,21]. In addition, TGFB stimulates not only cytokines but also prostaglandin-endoperoxide synthase 2 (PTGS2; cyclooxygenase-2—COX-2) encoded by the PTGS2 gene, which has been implicated in the pathogenesis of MDD [22,23]. Besides its role in inflammation, PTGS2 also catalyzes the conversion of arachidonic acid (AA) to prostaglandins (PGs), which further escalate inflammatory and neurodegenerative processes in CNS [24,25]. Importantly, research on an animal model of depression confirmed that PTGS2 levels are significantly elevated in various brain regions [26].

Another molecule strongly associated with inflammation is interferon regulatory factor 1 (IRF1). IRF1 was the first transcription factor identified in the interferon (IFN) system and plays a pivotal role in controlling the expression of many genes associated with the immune system [27]. IRF1 regulates IFN and other IFN-inducible genes involved in inflammation by influencing transcription [28]. Interferons, clusters of cytokines acting as signaling proteins in the immune response, play key roles in psychiatric conditions. For instance, IFN-α is an efficient stimulator of the proinflammatory cytokine network, including interleukin 1 (IL-1), interleukin 6 (IL-6) and tumor necrosis factor α (TNF-α), in both peripheral the CNS [9,29]; it is implicated in the adjustment of mood, sleep–wake cycle and behavior [29]. IRF1 also promotes the release of inflammatory cytokines and regulates the expression of interleukin 12 (IL-12) and interleukin 15 (IL-15), which are involved in MDD [28]. Furthermore, IRF1 interacts with several transcription factors, such as NF-kB [27].

Besides cytokines, various inflammatory pathways are thought to be dysregulated in MDD, including NF-kB, leading to increased levels of proinflammatory cytokines [30,31]. NF-kB is a ubiquitous transcriptional factor regulating the expression of genes involved in pleiotropic functions, including proinflammatory cytokines and costimulatory molecules [32,33,34]. In addition, NF-kB regulates neurogenesis and synaptic plasticity in the nervous system [35,36,37]. Moreover, some studies indicate the presence of an interplay between NF-kB and brain-derived neurotrophic factor (BDNF), which is a cornerstone of the neurotrophic hypothesis of depression [38,39]. More precisely, NF-kB can regulate BDNF expression and vice versa [40]. Normal NF-kB signaling is essential for neurogenesis, brain functioning, memory and neuronal plasticity [41,42]. Canonical signaling of NF-kB is activated by the IkB kinase (IKK complex), consisting of three subunits, one of which is IKK-B (inhibitor of nuclear factor kappa-B kinase subunit β) encoded by the IKBKB gene [43,44,45]. Therefore, alterations in IKBKB gene expression can disrupt the NF-kB system and may influence developing depression [43].

Despite the confirmed involvement of the immune system in depression, knowledge about inflammatory molecules other than cytokines is lacking. All selected genes contribute to neuroinflammation and brain functioning involved in the pathogenesis of depression. The occurrence of any variation in such genes may result in dysregulation and disruption of the other related factors. Moreover, the majority of them have not been studied in the context of psychiatric disorders yet. Therefore, all of these genes were selected to give a broader view of the inflammatory processes that may be activated in depression, not just focusing on cytokines, especially since all these factors are related to the regulation or stimulation of cytokine expression. Moreover, we have studied these genes in the context of the correlation of their single nucleotide polymorphisms (SNPs) with the risk of depression development. Therefore, the current study is a continuation of our research [46]. Stress is known to provoke inflammation in brain regions, such as the frontal cortex, hypothalamus and hippocampus, particularly sensitive to chronic stress [47,48]. Importantly, studies indicate an imbalance between pro- and anti-inflammatory cytokines in chronic mild stress (CMS)-induced depression [49]. It is hypothesized that antidepressant drug administration could effectively reduce proinflammatory cytokines in depressed subjects [50]. Selective serotonin reuptake inhibitors (SSRIs) and serotonin-norepinephrine reuptake inhibitors (SNRIs), such as venlafaxine, are currently used in the first-line treatment of MDD [51,52]. However, the chronic impact of antidepressants on the levels of inflammatory molecules in the peripheral and central nervous system has been barely studied. As it is only possible to directly study the brain of depressed patients post-mortem, therefore, such study requires using an animal model to understand the complex relationship between many processes, including the inflammation and etiology of MDD.

Therefore, the present study investigates whether: (1) the CMS procedure in rats, which closely mirrors depression in humans, can induce changes in TGFA, TGFB, IRF1, PTGS2 and IKBKB expression at the mRNA level in peripheral blood mononuclear cells (PBMCs) and in selected brain structures (hippocampus, amygdala, midbrain, hypothalamus, prefrontal cortex and basal ganglia); (2) chronic administration of serotonin-norepinephrine reuptake inhibitor, venlafaxine, alters the expression of these genes in the peripheral and central nervous system; (3) the CMS procedure and chronic venlafaxine administration cause epigenetic changes in the investigated genes, such as methylation level in the promoters; (4) the changes in expression observed in PBMCs can reflect similar changes in the brain.

## 2. Materials and Methods

### 2.1. Animals

Male Wistar Han rats, approximately 5 weeks old, weighing 200–220 g at the start (Charles River, Germany), were used to carry out the study. The animals were brought into the laboratory one month before the start of the experiment to adapt to the housing conditions. With the exceptions described below, the rats were housed singly with a maintenance 12 h light/dark cycle (lights on at 8.00) in controlled temperature (20 ± 2 °C) and humidity (50 ± 5%). Food and water were allowed ad libitum. All procedures used in the experiment were approved by the Bioethical Committee at the Institute of Pharmacology, Polish Academy of Sciences, Kraków, Poland, and conform to the rules and principles of Directive 86/609/ECC.

### 2.2. Chronic Mild Stress Procedure

Male Wistar Han rats were brought into the laboratory one month before the start of the experiment to adapt to the housing conditions. First, after acclimatization, the animals were trained to consume a 1% sucrose solution in baseline tests conducted once a week in the home cage. Sucrose solution consumption is the most common, adequate way to quantify the behavioral effect of CMS procedure by measuring the ability to respond to reward stimuli. It reflects the key symptom investigated in a depressed subject, which is anhedonia—inability to feel pleasure [53]. Sucrose solution was presented for one hour after 14 h water and food deprivation. Consumption of the sucrose was verified once a week, under controlled conditions, until the experiment was ended. Subsequently, based on their sucrose intakes in the final baseline test, the animals were divided into two matched groups. The control group (of nonstressed animals) was housed in separate rooms to exclude contact with the stressed animals. In this group, food and water were freely available, except for 14 h deprivation before each weekly sucrose test. The stressed group was exposed to the CMS procedure for a period of two or seven weeks. Each week of the stress regimen consisted of two periods of food and water deprivation, two periods of 45-degree cage tilt, two periods of intermittent illumination (light on and off every two hours), two periods of a soiled cage (250 mL water in sawdust bedding), one period of paired housing, two periods of low-intensity stroboscopic illumination (150 flashes/min), and three periods without stress. All stressors were applied for 10–14 h and were used individually and continuously, day and night. The rats subjected to the CMS procedure demonstrated a gradual decrease in sucrose solution consumption to approximately 40% of pre-stress values. After stabilization of this effect, named after two weeks of initial stress, the animals were either decapitated or further divided into matched subgroups and daily administrated with vehicle (1 mL/kg, IP) or venlafaxine (10 mg/kg, IP) for the subsequent five weeks. The drug was administrated to both control and stressed animals. The weekly sucrose tests were carried out 24 h after the last dose. After the final sucrose test, i.e., after seven weeks of stress, or rather, the completion of five-week administration of vehicle or drug, the animals were decapitated, and blood and brain samples were collected. Before decapitation, no anesthesia was used to avoid possible changes in the expression of genes in the brain caused by the anesthetic. The detailed description of stressors and CMS schedule are presented in Table 1.

### 2.3. Specimen Collection

Peripheral blood mononuclear cells (PBMCs) were isolated from blood samples collected into 5 mL vacutainers with EDTA. Isolation was based on differential migration of cells during centrifugation. Precisely, blood was mixed with equal volumes of PBS, layered on top of Gradisol L (Aqua-Med, Lodz, Poland) and centrifuged. The interfacial layer (lymphocyte coat) was transferred to a new tube and centrifuged. The supernatant was removed, and PBMCs stored as pellets at −20 °C until used.

### 2.4. RNA and DNA Isolation from Peripheral Blood Mononuclear Cells

RNA and DNA isolation was performed using the commercial spin column methods with elution in RNAse-Free water (GenElute mammalian total RNA miniprep kit, Sigma-Aldrich, St. Louis, MO, USA; QIAamp DNA mini kit, Qiagen, Hilden, Germany, respectively), following the manufacturer’s instructions. Total DNA and RNA concentrations were determined spectrophotometrically. The purity of samples was measured as 260/280 nm OD ratio with expected values of 1.8–2.0. RNA and DNA samples were stored at −20 °C until further analysis.

### 2.5. Specimen Collection; RNA and DNA Isolation from Brain Tissues

Brain regions, i.e., hippocampus, amygdala, midbrain, hypothalamus, prefrontal cortex and basal ganglia, were separated and immediately frozen in liquid nitrogen and stored at −80 °C. In the isolation procedure, a sufficient volume of PBS was added to each sample and then homogenized using FastGene^®^ tissue grinder (Nippon Genetics Europe, Düren, Germany). The homogenized samples were then sonicated, centrifuged and rinsed with PBS by a commercial kit (ISOLATE II RNA/DNA/protein kit; Bioline), according to the manufacturer’s protocol. The purity of the RNA and DNA and their concentrations were measured spectrophotometrically by calculating the ratio between absorbance at 260 nm and 280 nm. Samples were stored at −20 °C until further analysis.

### 2.6. Reverse Transcription and Gene Expression

The reverse transcription reaction was performed with the use of a high-capacity cDNA reverse transcription kit (Applied Biosystems, Foster City, CA, USA). The total reaction volume was 20 μL. The mixture contained nuclease-free water, 10xRT buffer, 10xRT random primers, 25xdNTP Mix (100 mM), total RNA (0.5 ng/μL) and MultiScribe^®^ reverse transcriptase. The reaction tubes were incubated for 10 min at 25 °C, 120 min at 37 °C, and then for 5 min at 85 °C to inactivate the reverse transcriptase. PCR was performed in a C1000™ programmed thermal cycler (Bio-Rad Laboratories Inc., Hercules, CA, USA). After the reaction, the cDNA samples were stored at −20 °C. TaqMan gene expression assay (Thermo Fisher Scientific, Waltham, Massachusetts, USA) was used to examine the expression of the following genes: *IKBKB* (assay ID: Rn00584379_m1), *TGFA* (assay ID: Rn00446234_m1), *TGFB* (assay ID: Rn00572010_m1), *IRF1* (assay ID: Rn01483828_m1), *PTGS2* (assay ID: Rn01483828_m1). The reaction was performed using CFX96™ real-time PCR detection system thermal cycler (Bio-Rad Laboratories Inc., Hercules, CA, USA). The housekeeping gene 18S ribosomal RNA gene (18S) (assay ID: Hs99999901_s1) was applied as an internal control (reference gene) for all reverse transcription–quantitative polymerase chain reactions (RT–qPCR). The reaction mixture contained the following: cDNA samples, a TaqMan Universal master mix, no UNG (Applied Biosystems, Foster City, CA, USA), TaqMan probe (Thermo Fisher Scientific, Waltham, Massachusetts, USA) and RNAse-free water. The PCR protocol was as follows: 10 min at 95 °C (enzyme activation), followed by 60 cycles of 30 s at 95 °C (denaturation), and one minute at 60 °C (for annealing/extension). The cycle threshold (Ct) values were calculated automatically by a CFX96 real-time PCR detection system software System (Bio-Rad Laboratories Inc., Hercules, CA, USA). For each sample, the gene expression of the target mRNA was calculated relative to a reference gene (ΔCt sample = Ct target gene − Ct reference gene). The levels of gene expression are given as a normalization ratio calculated as fold = 2 − ΔCt sample.

### 2.7. Methylation and HRM Analysis

The methylation status of investigated gene promoters was obtained by methylation-sensitive high-resolution melting [54,55]. Genes sequences were checked for the numbers of promoters and the presence of CpG islands. The promoter sequence was obtained from the Eukaryotic promoter database EPD (http://epd.vital-it.ch (accessed on 1 December 2018)) [56]. For all investigated genes, the region from −499 to 100 bp relative to the transcription start site (TSS) was used to design primers. The selected region contains all core promoter motifs, required CpG island as well, as is characterized by the presence of curved DNA elements relevant to the transcription process. Primers were designed for promoters containing CpG islands using Methyl Primer Express™ Software v 1.0 (Thermo Fisher Scientific, Waltham, Massachusetts, USA) according to recommendations provided by Wojdacz et al. (2009) [57]. It was not possible to design suitable MS-HRM primers for TGFB (Table 2). The bisulfite conversion reaction was performed using 200 ng of DNA with a CiTi converter DNA methylation kit (A&A Biotechnology, Gdynia, Poland), according to the manufacturer’s instruction. Methylated DNA (CpGenome™ rat methylated genomic DNA standard; Merck Millipore, Burlington, MA, USA) and unmethylated DNA (CpGenome™ rat unmethylated genomic DNA standard; Merck Millipore, Burlington, MA, USA) were used as controls for the MS-HRM experiments. To maintain accuracy and control the sensitivity of methylation detection, a series of dilutions were prepared, namely: nonmethylated, 10% methylated, 25% methylated, 50% methylated, 75% methylated, and 100% methylated DNA. These reactions were performed using the Bio-Rad CFX96 real-time PCR detection system and analyzed in HRM Powered by Precision Melt Analysis™ software (Bio-Rad Laboratories Inc., Hercules, CA, USA). Each reaction mixture contained 5× HOT FIREPol^®^ EvaGreen^®^ HRM Mix (no ROX) (Solis BioDyne, Tartu, Estonia), 500 nM of each primer and 10 ng of bisulfite converted DNA (theoretical calculation). The parameters for amplification and HRM analyses included initial activation for 12 min at 95 °C, 45 cycles of 95 °C for 15 s; annealing at optimal primer temperatures (tested experimentally) for 20 s and elongation at 72 °C for 20 s. The HRM analysis consisted of denaturation at 95 °C for 15 s, reannealing at 60 °C for one minute and melting from 60 to 95 °C at a ramp rate of 0.2 °C.

### 2.8. Drugs

Venlafaxine HCl (Carbosynth Ltd., Compton, Berkshire, UK) was dissolved in 0.9% sterile saline, which was used for vehicle administration. The drug was then administered IP at a volume of 1 mL/kg of body weight, i.e., a dose of 10 mg/kg, as used previously [58,59].

### 2.9. Statistical Analysis

The effect of initial two-week stress on sucrose consumption was analyzed by *t-*test for normally distributed data or the Mann–Whitney rank-sum test for non-normally distributed data. In addition, when the data were normally distributed, the sucrose intake, gene expression and methylation data were analyzed using one-way analysis of variance (one-way ANOVA), with Tukey’s test as a post hoc test; F ratios were significant for the groups’ control/vehicle, stressed/vehicle and stressed/venlafaxine. If the data were not normally distributed, these relationships were tested using the Kruskal–Wallis one-way ANOVA on ranks, followed by post hoc Student–Newman–Keuls test. The student’s t-test was used to analyze differences between blood and brain samples. *p* values < 0.05 were considered significant. Analyses were performed using Statistica 12 (StatSoft, Tulsa, OK, USA), SigmaPlot 11.0 (Systat Software Inc., San Jose, CA, USA) and GraphPad Prism 5.0 (GraphPad Software, Inc., La Jolla, CA, USA).

## 3. Results

### 3.1. Sucrose Intakes and Body Weights of Animals Exposed to CMS and Venlafaxine Administration

The 1% sucrose solution intake was comparable in all groups before CMS procedure initiation (week 0). Following the initial two-week stress, the consumption decreased to approximately 60% of initial values (week 2; stressed). Intakes remained at low levels in stressed animals administered with the vehicle until the end of the experiment (week 7; stressed/saline). Although chronic (five-week) venlafaxine treatment yielded no effect in control animals, it normalized sucrose consumption in stressed rats (Table 3). Both stress and venlafaxine had no significant effect on the body weights of the control or CMS animals (Table S1, Supplementary Materials).

### 3.2. Gene Expression

#### 3.2.1. Gene Expression in PBMCs after CMS Procedure and Venlafaxine Administration

**Figure 1:**
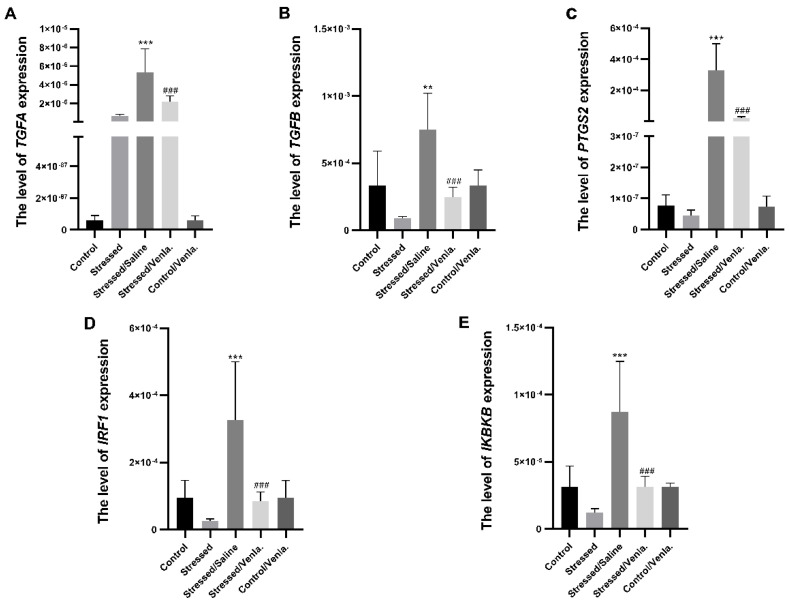
mRNA expression of TGFA (**A**), TGFB (**B**), PTGS2 (**C**), IRF1 (**D**) and IKBKB (**E**) in PBMCs of animals exposed to chronic mild stress (CMS) for two weeks (control, stressed) and in animals exposed to CMS for seven weeks and administered vehicle (1 mL/kg) or venlafaxine (10 mg/kg) for five weeks (stressed/saline, stressed/venlafaxine, control/venlafaxine). Relative gene expression levels were estimated using the 2−ΔCt (Ct gene–Ct 18S) method. Data represent means ± SD. N = 6; ** *p* < 0.01; *** *p* < 0.001 relative to control group; ### *p* < 0.001 relative to stressed/saline group.

The mRNA expression level of TGFA, TGFB, PTGS2, IRF1 and IKBKB in PBMCs did not differ between the control and stressed groups for the initial two weeks. However, animals stressed for seven weeks and administered saline demonstrated significantly greater expression of all studied genes compared to the control group, i.e., TGFA (F = 22.027, df = 4, *p* < 0.001, Tukey’s test *p* < 0.001), TGFB (F = 11.383, df = 4, *p* < 0.001, Tukey’s test *p* < 0.001), PTGS2 (F = 20.803, df = 4, *p* < 0.001, Tukey’s test *p* < 0.001), IRF1 (F = 11.239, df = 4, *p* < 0.001, Tukey’s test *p* < 0.001), IKBKB (F = 13.817, df = 4, *p* < 0.001, Tukey’s test *p* < 0.001). Chronic treatment with venlafaxine (five weeks) yielded no effect in control animals, but caused a significant decrease in the expression of all studied genes in stressed rats, i.e., TGFA (F = 22.027, df = 4, *p* < 0.001, Tukey’s test *p* < 0.001), TGFB (F = 11.383, df = 4, *p* < 0.001, Tukey’s test *p* < 0.001), PTGS2 (F = 20.803, df = 4, *p* < 0.001, Tukey’s test *p* < 0.001), IRF1 (F = 11.239, df = 4, *p* < 0.001, Tukey’s test *p* < 0.001), IKBKB (F = 13.817, df = 4, *p* < 0.001, Tukey’s test *p* < 0.001) (Figure 1).

#### 3.2.2. Gene Expression in Brain Structures after CMS Procedure and Venlafaxine Administration

**Figure 2:**
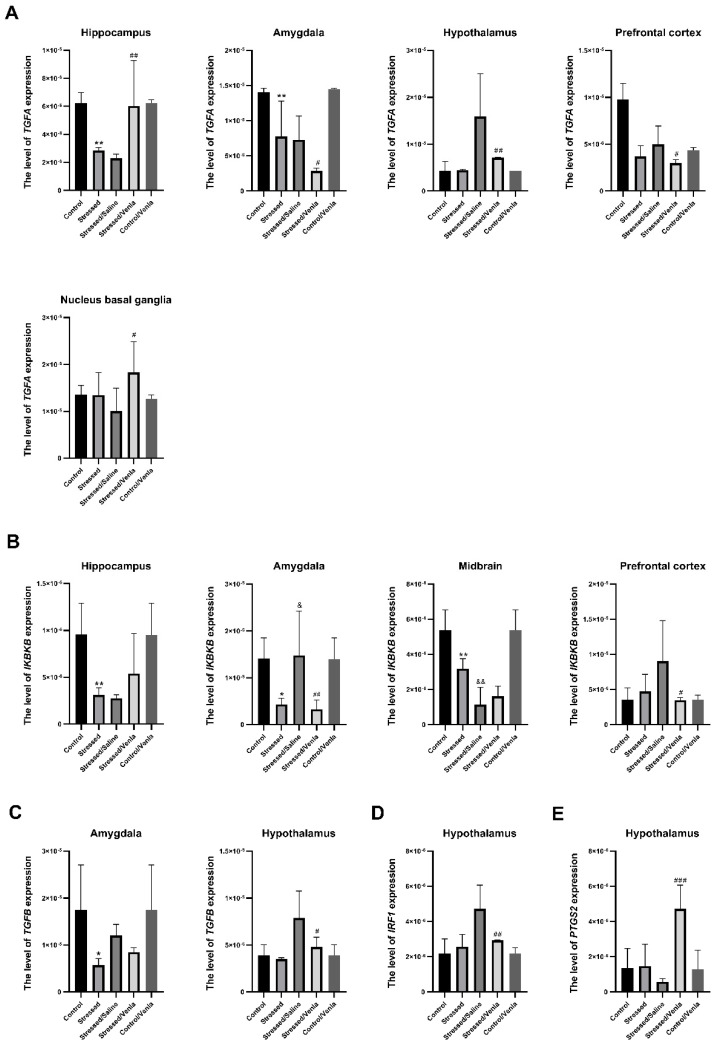
mRNA expression of TGFA (**A**), IKBKB (**B**), TGFB (**C**), IRF1 (**D**) and PTGS2 (**E**) in the brain structures of animals exposed to chronic mild stress (CMS) for two weeks (control, stressed) and in animals exposed to CMS for seven weeks and administered vehicle (1 mL/kg) or venlafaxine (10 mg/kg) for five weeks (control/venlafaxine, stressed/saline, stressed/venlafaxine). Relative gene expression levels were estimated using a 2−ΔCt (Ctgene–Ct18S) method. Data represent means ± SD. N = 6. *p < 0.05; **p < 0.01 relative to control group. # *p* < 0.05; ## *p* < 0.01; ### *p* < 0.001 relative to stressed/saline group. & *p* < 0.05; && *p* < 0.01 relative to stressed group.

The effect of CMS and antidepressant administration on the mRNA expression of the studied genes clearly differed between brain structures. All statistically significant results are shown in Figure 2. The two-week CMS caused a significant decrease of TGFA (F = 10.364, df = 4, *p* < 0.001, Tukey’s test *p* = 0.006), and IKBKB (F = 7.985, df = 4, *p* < 0.001, Tukey’s test *p* = 0.006) expression in the hippocampus. Furthermore, stress induced lower expression of TGFA (F = 19.543, df = 4, *p* < 0.001, Tukey’s test *p* = 0.004), TGFB (F = 4.408, df = 4, *p* = 0.008, Tukey’s test *p* = 0.022) and IKBKB (F = 7.311, df = 4, *p* < 0.001, Tukey’s test *p* = 0.024) in the amygdala, and in the midbrain in the case of IKBKB (F = 27.746, df = 4, *p* < 0.001, Tukey’s test *p* = 0.004). Interestingly, this effect was intensified in animals after the seven-week CMS procedure. After venlafaxine administration, the stressed animals demonstrated downregulation of TGFA (F = 8.635, df = 4, *p* < 0.001, Tukey’s test *p* < 0.001), TGFB (F = 8.058, df = 4, *p* < 0.001, Tukey’s test *p* < 0.001) and IRF1 (F = 10.804, df = 4, *p* < 0.001, Tukey’s test *p* < 0.001) in the hypothalamus, IKBKB (F = 4.029, df = 4, *p* = 0.012, Tukey’s test *p* = 0.024) levels in the prefrontal cortex and IKBKB (F = 7.311, df = 4, *p* < 0.001, Tukey’s test *p* < 0.015) in the amygdala. On the other hand, venlafaxine treatment also increased the expression of TGFA in the hippocampus (F = 10.364, df = 4, *p* < 0.001, Tukey’s test *p* = 0.002) and nucleus basal ganglia (F = 2.815, df = 4, *p* = 0.047, Tukey’s test *p* = 0.024), as well as PTGS2 level in the hypothalamus (F = 13.733, df = 4, *p* < 0.001, Tukey’s test *p* < 0.001). Furthermore, no differences in mRNA expression level were found after venlafaxine administration in the nonstressed control group (Figure S1, Supplementary Materials).

### 3.3. Methylation of Studied Genes Promoters

#### 3.3.1. Methylation Status in PBMCs after CMS Procedure and Venlafaxine Administration

**Figure 3:**
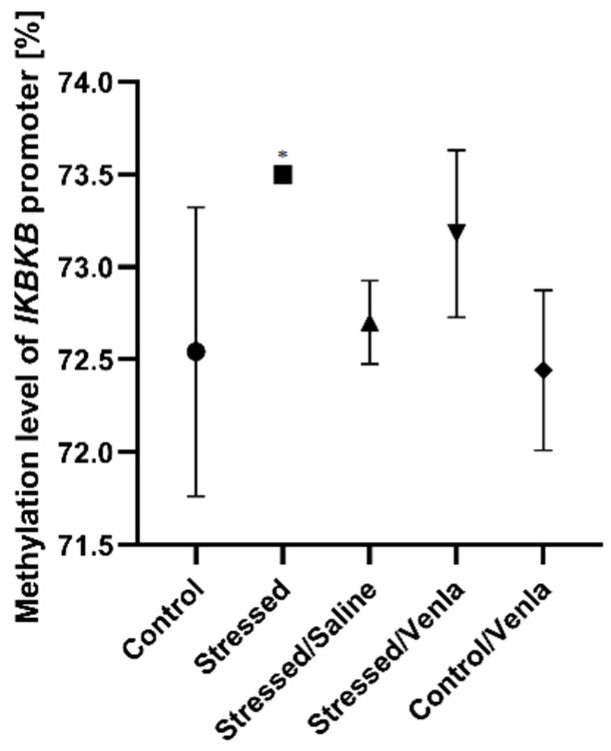
Methylation level of IKBKB promoter in PBMCs of animals exposed to chronic mild stress (CMS) for two weeks (control, stressed) and in animals exposed to CMS for seven weeks, including five-week administration of vehicle (1 mL/kg) or venlafaxine (10 mg/kg) (control/venlafaxine, stressed/saline, stressed/venlafaxine). Data represent means ± SD. N = 6. * *p* < 0.05 relative to control group.

The only significant change in methylation status was found in the case of the IKBKB promoter (Figure 3), where two-week exposure to CMS caused increased methylation compared with nonstressed controls (F = 5.777, df = 4, *p =* 0.002, Tukey’s test *p =* 0.011). No significant differences were observed for promoters of other investigated genes in PMBCs.

#### 3.3.2. Methylation Status in Brain after CMS Procedure and Venlafaxine Administration

**Figure 4:**
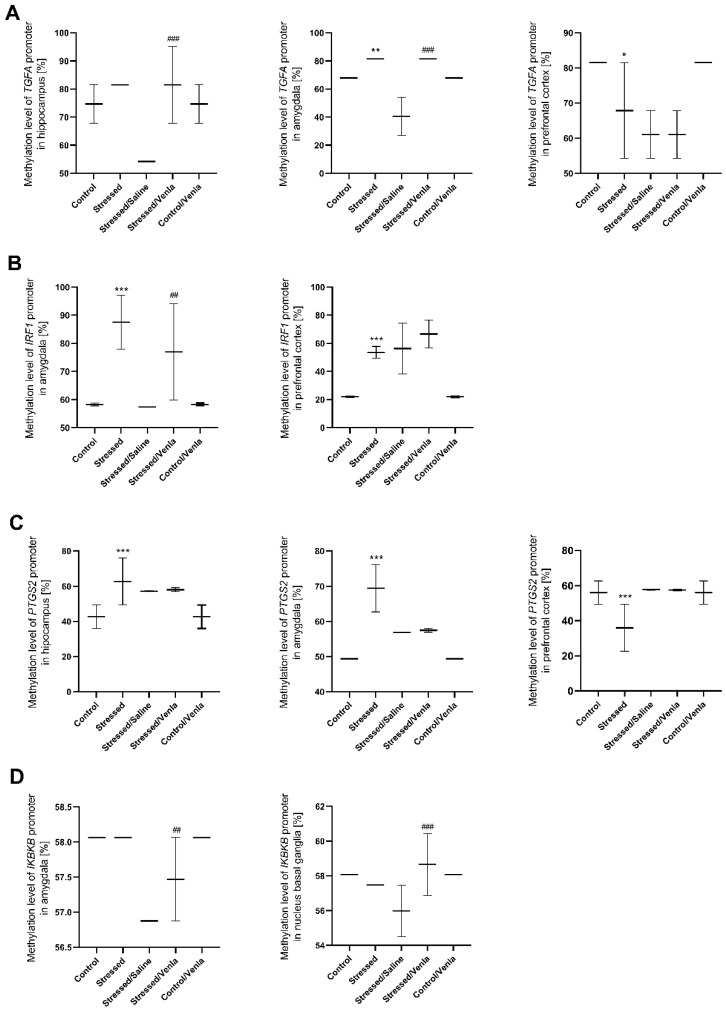
Methylation levels of the TGFA (**A**), IRF1 (**B**), PTGS2 (**C**) and IKBKB (**D**) promoter in brain regions of animals exposed to chronic mild stress (CMS) for two weeks (control, stressed) and in animals exposed to CMS for seven weeks, including five-week administration of vehicle (1 mL/kg) or venlafaxine (10 mg/kg) (control/venlafaxine, stressed/saline, stressed/venlafaxine). Data represent means ± SD. N = 6. * *p* < 0.05, ** *p* < 0.01, *** *p* < 0.001 relative to control group. ## *p* < 0.01 ###; *p* < 0.001 relative to stressed/saline group.

All statistically significant results are shown in Figure 4. CMS procedure significantly increased the methylation level of the TGFA promoter in the amygdala (F = 45.000, df = 4, *p* < 0.001, Tukey’s test *p* = 0.006). Stressed animals also demonstrated a higher methylation status in the case of the IRF1 promoter in the amygdala (F = 14.765, df = 4, *p* < 0.001, Tukey’s test *p* < 0.001) and prefrontal cortex (F = 29.138, df = 4, *p* < 0.001, Tukey’s test *p* < 0.001), as well as in the case of the PTGS2 promoter in the hippocampus (F = 9.749, df = 4, *p* < 0.001, Tukey’s test *p* < 0.001) and amygdala (F = 44.933, df = 4, *p* < 0.001, Tukey’s test *p* < 0.001). However, CMS also caused a decrease in PTGS2 (F = 9.777, df = 4, *p* < 0.001, Tukey’s test *p* < 0.001) as well as TGFA (F = 12.000, df = 4, *p* < 0.001, Tukey’s test *p* = 0.003) promoter methylation in the prefrontal cortex Interestingly, chronic five-week administration of venlafaxine resulted in increased IKBKB promoter methylation in the amygdala (F = 24.000, df = 4, *p* < 0.001, Tukey’s test *p* < 0.001) and nucleus basal ganglia (F = 5.803, df = 4, *p* = 0.002, Tukey’s test *p* < 0.001), and the IRF1 promoter in the amygdala (F = 14.765, df = 4, *p* < 0.001, Tukey’s test *p* = 0.006). A similar effect was observed in the case of the TGFA promoter, where the methylation status was higher in the hippocampus (F = 13.500, df = 4, *p* < 0.001, Tukey’s test *p* < 0.001) and amygdala (F = 45.000, df = 4, *p* < 0.001, Tukey’s test *p* < 0.001). No other differences in mRNA expression level were found (Figure S2, Supplementary Materials).

## 4. Discussion

The present study is the first to investigate the levels of TGFA, IRF1 and IKBKB mRNAs in an animal model of depression. The study also briefly examined the influence of other genes, such as PTGS2 and TGFB, and the effect of prior venlafaxine treatment on depression as knowledge about their role in the etiopathomechanism of depressive disorders is lacking; this is particularly important as all these factors play roles in neuroinflammatory processes and brain functioning. Moreover, any variation in one of the results in dysregulation and disruption in the others. Therefore, all of these genes were investigated to give a broader view of the inflammatory processes that may be activated in depression, not just focusing on cytokines.

Our study is the first to describe the effect of CMS [53] on the expression of selected genes in the PBMCs and six brain regions (hippocampus, amygdala, hypothalamus, midbrain, prefrontal cortex and basal ganglia). They also present the impact of chronic administration of venlafaxine on the mRNA level in this context. The results are also enriched with a study of whether these factors can induce epigenetic changes, i.e., the methylation status of the gene promoters studied, in blood and brain samples. Our results indicate that for the initial two weeks, the mRNA expression of TGFA, TGFB, PTGS2, IRF1 and IKBKB in PBMCs did not differ between the control and stressed groups. However, after longer exposure to CMS, i.e., for a subsequent five weeks, the expression of all studied genes was significantly upregulated. It might suggest that activation of inflammatory pathways in the periphery could only be triggered after longer exposure to chronic stress conditions.

Our results regarding TGFB are consistent with those of other animal studies demonstrating its increased expression in mice subjected to depression induced by unpredictable mild stress [19]. On the other hand, other studies suggest a significant blood TGFB level is significantly lower in depressed patients compared to healthy controls [20,21,60,61]. Interestingly, we observed that expression of TGFB was significantly diminished in the amygdala after CMS procedure and that chronic (five-week) treatment with venlafaxine caused a decrease of TGFB expression in the PBMCs and the hypothalamus of stressed rats. This observation contradicts previous findings concerning antidepressant drugs; more precisely, it has been reported that treatment with antidepressants caused up-regulation of TGFB in plasma [61,62]. It has been proposed that its protein product plays a role in maintaining the stability of immunologically privileged sites, such as the central nervous system. In addition, as TFGB plays a complex role in stimulating the production of various cytokines [63], it is possible that using antidepressants, including venlafaxine, may change the balance between pro- and anti-inflammatory cytokines by changing the levels of TGFB in depression. It is, therefore, also possible that increased expression of TGFB in chronic stress conditions may occur in response to the elevated levels of proinflammatory agents commonly found in depression. Another investigated gene in the TGF family, TGFA, encodes polypeptide growth factor. Both genes regulate embryonic development and immune response [14,15]. In the present study, low expression of TGFA was observed in the PBMCs of control animals; however, this was significantly upregulated after CMS; interestingly, the TGFA mRNA levels were higher in the hippocampus and amygdala of the nonstressed group than the CMS rats. In the case of the hippocampus, this effect was normalized after venlafaxine administration. Similarly, in PMBCs, antidepressant therapy led to downregulation of TGFA expression, reversing the effects of the CMS procedure. In addition, lower TGFA expression was observed after venlafaxine administration in the amygdala, hypothalamus and prefrontal cortex. Interestingly, CMS caused increased TGFA promoter methylation in the amygdala, which could be associated with lower levels of TGFA expression. In addition, higher levels of methylation were observed after antidepressant therapy, which could be connected with the downregulation of TGFA expression observed in the amygdala of rats treated with venlafaxine. This is the first set of such results concerning the role of TGFA in depression or other psychiatric disorders. However, it has been found to play a role in the induction of proliferation and differentiation of neural cells in the adult mammalian brain: exogenous TGFA administration was observed to trigger repair mechanisms after nervous system injury and to have neuroprotective properties against cytotoxic and apoptotic signals [64]. It is hypothesized that TGFA could improve or the state of neurodegenerative disorders, such as Parkinson’s disease, as well as post-traumatic and stroke brain injury, and even reverse some of their characteristic features. Therefore, future studies should consider the possible role of TGFA in psychiatric disorders. Another gene believed to be associated with mechanisms of depression is PTGS2. It is widely accepted that PTGS2 participates in inflammatory processes partly involved in neurodegeneration in the CNS. PTGS2 and its downstream product PGs play important roles in triggering an inflammatory cascade in depression [24,25]. Our results indicate that its expression was significantly upregulated in PBMCs after seven-week chronic stress, and this effect was, at least partially, reversed by chronic venlafaxine administration. In the case of brain tissues, we only observed one significant change after the antidepressant treatment: a higher level of PTGS2 mRNA in the hypothalamus. We also observed that the CMS procedure increased PTGS2 promoter methylation in the hippocampus and amygdala and reduced it in the prefrontal cortex. However, this methylation pattern seemed to be unrelated to PTGS2 expression. Our findings regarding the PTGS2 mRNA expression gene are consistent with previous reports indicating significantly increased levels in the peripheral blood cells of depressed patients versus healthy controls [65]. Furthermore, in a model depression in adult rats caused by neonatal treatment with the antidepressant drug clomipramine, PTGS2 mRNA expression was increased in the hippocampus. At the same time, the protein level was elevated in the entorhinal cortex, and that the usage of NSAID PTGS2 inhibitors, i.e., COX-2 inhibitors, could reverse depressive behavior [26]. This is in line with another study proving that chronic unpredictable mild stress caused increased PTGS2 expression accompanied with depressive symptoms, which was further neutralized by PTGS2 RNAi lentivirus inhibitor [66]. Administration of COX2 selective inhibitor in depressed rats has also been found to reduce depressive behavior and diminish the levels of cytokines in the hypothalamus of rats [67]. Moreover, for patients with severe depression, therapy with a selective inhibitor cannot only alleviate depressive behavior but also reduce the serum level of proinflammatory cytokines [68]. The upregulation of the PTGS2 gene observed in the course of depression, together with the effectiveness of its inhibitors in therapy, confirm that PTGS2 plays a role in developing depressive disorders. Furthermore, our findings also suggest that venlafaxine has anti-inflammatory activity. The present research examined whether the expression of IRF1 changes during a depression-like state since it plays a pivotal role in controlling the expression of a number of genes whose products are essential in immunity [27]. As stated, IRF1 regulates the transcription of IFN and other IFN-inducible genes, all of which play a role in inflammation [28]. In the present study, IRF1 mRNA level was found to be significantly increased in the PMBCs of rats exposed to CMS; however, this fell to around control levels after venlafaxine administration. Venlafaxine treatment also lowered IRF1 expression in the hypothalamus. Regarding epigenetics, the CMS procedure resulted in an increase of IRF1 promoter methylation in the amygdala and prefrontal cortex, while antidepressant treatment caused higher IRF1 methylation in the amygdala. However, these changes did not affect the mRNA expression of the gene. This may suggest that other processes have a greater impact on the expression of this gene than the methylation of promoter sequences. To date, there has been no research regarding the role of IRF1 in depression and other psychiatric disorders, nor the level of its expression in these conditions. However, we could hypothesize that stress causes increased IRF1 expression and thus increased activation of inflammatory pathways, which is commonly observed in the course of depression. In addition, disruptions in NF-kB signaling, commonly observed in MDD, result in increased levels of proinflammatory cytokines. NF-kB regulates the expression of various genes involved in the immune response [30,31]. One of the IkB kinase subunits, IKKB, encoded by the IKBKB gene, is known to regulate NF-kB activity [43]. Studies have suggested that chronic unpredictable mild stress (CUMS) induces an increase of IKKB protein levels in the hippocampus [69]; however, our present findings indicate that CMS reduced IKBKB mRNA levels in the hippocampus, as well as in the amygdala and midbrain. In addition, in contrast to the brain, CMS resulted in increased IKBKB mRNA expression in PMBCs, which was decreased by venlafaxine treatment. Therefore, it is possible that inhibition of IKKB- NF-kB signaling pathways may exert an antidepressant-like effect and silence the neuroinflammation [69,70]. As the activation of NF-kB signaling promotes the release of proinflammatory cytokines, increased mRNA expression of IKBKB in PMBCs, which acts as a regulatory factor for NF-kB, could contribute to the activation of inflammatory pathways in blood cells; however, this effect is not reflected in the brain. These differences between tissue types may be associated with their response to stress stimuli. However, it is worth adding that the fact that an elevated level of IKBKB is not associated with high promoter methylation status. The change in promoter methylation status is low enough (approximately 1%) that despite its statistical significance, it may not be biologically relevant. Moreover, it could also suggest that other forms of expression regulation may have a greater influence.

Our findings are mostly in line with those of previous reports and support the concept that depressive disorders accompany alterations of multiple aspects of the immune response, both in the peripheral nervous system and in the central nervous system. Our work has some limitations, particularly a lack of protein level analysis. However, such an examination could not be performed in this study due to material limitations. Moreover, obtained results were characterized by a wide variability between different parts of the brain and between blood and brain samples. It is worth mentioning that in most cases, the investigated genes demonstrated significantly higher expression in blood than brain tissues; however, it can only be speculated whether this is due to a distinct tissue response or other factors. The promoter methylation changes, despite being statistically significant, are not always reflected by altered expression patterns. This suggests that these changes have not been biologically relevant or/and other factors may have a greater influence on expression regulation. Mainly, expression changes are controlled by methylation status. However, other epigenetic modifications, such as modification of histones and microRNAs, could be implicated [71]. Additionally, discordant changes in methylation and expression patterns may be dependent on methylation changes in other cytosines, either outside investigated regions or associated with non-CpG sites. Moreover, other variables that must be taken into consideration are sequences recognized by methylation-sensitive transcriptional factors. In this case, even single methylated or unmethylated cytosine influence the affinity of the TF, and therefore, impact the gene expression. However, this phenomenon is still not well-known, as well as the list of the potentially methylation-sensitive TF is continuously changing [72]. Therefore, it is an interesting perspective research area. MS-HRM analysis could have some potential limitations, such as primer competition, finding suitable primer binding sites as well as issue of the PCR bias. However, all the imperfections of the method can be minimized or even eliminated following the rules carefully [57,73]. As mentioned, MS-HRM analysis has some limitations. In our study, it was not possible to find suitable primer-binding sites in sequences with high CpG content and thus to design primers for the TGFB gene. Additionally, it is worth adding that non-CpG sites, which have not been analyzed in our study, might be differentially methylated and thus affect gene expression [74]. Therefore, observed results should be extrapolated with caution. It is also difficult to develop a single stable and faultless animal model of depression, particularly since many human symptoms cannot be modeled in laboratory animals. We used a validated CMS animal model, which closely mirrors depression in humans. However, it should be remembered that it is based only on anhedonia, reflected by reduced sucrose intake [53]. Moreover, daily injections of drugs or vehicles may act as an additional stress factor of the CMS. However, we believe that such research moves one step closer to the possibility of conducting research on patients.

## 5. Conclusions

Our main findings indicate that the TGFA, TGFB, PTGS2, IRF1 and IKBKB genes could be responsible for activating inflammatory pathways after stress stimuli. More precisely, this research confirms that CMS is associated with changes in the mRNA expression of these genes, both in PMBCs and regions of the brain, which in turn could trigger an inflammatory cascade. Another key finding is the fact that chronic administration of venlafaxine may cause anti-inflammatory effects by affecting the expression of the investigated genes. Furthermore, both CMS and venlafaxine administration caused changes in promoter methylation status. However, contradictory results in this area suggest that other epigenetic mechanisms could play a significant role in the expression regulation of the aforementioned genes. The results also indicate that individual brain structures demonstrate different tissue responses for stress and antidepressant drugs, suggesting that reactions are region-specific. Nevertheless, our findings confirm at least a partial association between TGFA, TGFB, PTGS2, IRF1 and IKBKB genes and depression, and hence it is highly likely that inflammation plays a role in psychiatric disorders. Such observations serve as a further step towards understanding the underlying processes of depression and the mechanisms of action of antidepressants.

## Figures and Tables

**Table 1:** Schedule of CMS procedure and detailed description of all applied stressors.

Experiment Start				
5 weeks adaptation to 1% sucrose consumption test				
2 weeks without stress	2 weeks of initial stress			
5 weeks without stress and with venlafaxine administration	5 weeks of stress with saline administration		5 weeks of stress with venlafaxine administration	
	**Stress Procedure**			
	Stressor	Duration		Number of periods
	Food and water deprivation	10–14 h		2 periods
	45-degree cage tilt	10–14 h		2 periods
	Soiled cage (250 mL water in sawdust bedding)	10–14 h		2 periods
	Paired housing	10–14 h		1 period
	Low-intensity stroboscopic illumination (150 flashes/min)	10–14 h		2 periods
	Intermittent illumination	10–14 h (light on and off every two hours)		2 periods
	No stress	10–14 h		3 periods
Final sucrose consumption test and decapitation				

**Table 2:** The specification of primers used for the analysis of methylation levels in the promoter regions of the studied genes.

Gene	Starter Sequence (5′->3′)	Tm (°C)	Product Size (bp)	Number of CpG Islands	Product %CGs	CpGs in Product
IKBKB	F:AGGGTGGTTTTTTATTTTTATTTT R:AACCCCCACTAAAACTAACTTAA	55	117	1	36.75	5
IRF1	F:TTGGAGATTTAGGGAGTTAGGT R:CCCCTTACCTATCTTAAAAAACC	55	123	1	43.90	4
PTGS2	F:GTAATAGTAGGGAGGAAAAATTTTAA R:ATCCTAACAAACCCCAAA	55	111	1	37.84	10
TGFA	F:GTTTTTTTAGGTGGTTGGTTAAG R:CTTCAAACACCTCCCTACAATA	55	188	1	42.55	11

**Table 3:** Sucrose intakes in animals exposed to chronic mild stress (CMS) for two weeks and in animals exposed to CMS or venlafaxine.

Weeks of CMS	Control	Stressed	Stressed/Saline	Stressed/Venlafaxine	Control/Venlafaxine
Week 0	12.6 ± 1.6	11.0 ± 0.7	11.7 ± 0.7	11.4 ± 0.5	11.9 ± 0.7
Week 2	15.6 ± 1.9	6.8 ± 1.0 **	4.9 ± 0.6 ****	5.8 ± 0.5 *	13.9 ± 0.9
Week 7	-	-	6.1 ± 0.7	12.6 ± 1.0 ***	13.3 ± 1.3

Data represent means ± SEM. N = 6. ** *p* < 0.01; relative to week 0 in the stressed group. * *p* < 0.05; relative to week 0 in the stressed/venlafaxine group. *** *p* < 0.01; relative to week 2 in the stressed/venlafaxine group. **** *p* < 0.001; relative to week 0 in the stressed/saline group.

## Data Availability

The data that support the findings of this study are available from the corresponding author (T.S.) upon responsible request.

## Supplementary Materials

The following are available online at https://www.mdpi.com/article/10.3390/genes12050667/s1, Table S1: The effect of CMS procedure and venlafaxine on the body weights of the animals; Figure S1: mRNA expression of TGFA (A), IKBKB (B), TGFB (C), IRF1 (D) and PTGS2 (E) in the brain structures of animals exposed to chronic mild stress (CMS) for two weeks (control, stressed) and in animals exposed to CMS for seven weeks and administered vehicle (1 mL/kg) or venlafaxine (10 mg/kg) for five weeks (control/venlafaxine, stressed/saline, stressed/venlafaxine). Relative gene expression levels were estimated using a 2−ΔCt (Ctgene–Ct18S) method. Data represent means ± SD. N = 6.; Figure S2: Methylation levels of the TGFA (A), IRF1 (B), PTGS2 (C) and IKBKB (D) promoter in brain regions of animals exposed to chronic mild stress (CMS) for two weeks (control, stressed) and in animals exposed to CMS for seven weeks, including five-week administration of vehicle (1 mL/kg) or venlafaxine (10 mg/kg) (control/venlafaxine, stressed/saline, stressed/venlafaxine). Data represent means ± SD. N = 6.
