# Supplementary material for: Chronic Mild Stress and Venlafaxine Treatment Were Associated with Altered Expression Level and Methylation Status of New Candidate Inflammatory Genes in PBMCs and Brain Structures of Wistar Rats
Source: Genes (Basel). 2021 Apr 29;12(5):667. doi: 10.3390/genes12050667 (PMC8146372; doi:10.3390/genes12050667)
Supplement: Supplementary file 1 [file genes-12-00667-s001.zip › Table S1.pdf]

Table S1. The effect of CMS procedure and venlafaxine on the body weights of the animals

Group	Treatment	Weeks	Body weights (g)	Weeks	Body weights (g)	Weeks	Body weights (g)
Control	No treatment	Baseline	335	Week 2	345		
Control	No treatment	Baseline	350	Week 2	365		
Control	No treatment	Baseline	375	Week 2	385		
Control	No treatment	Baseline	335	Week 2	345		
Control	No treatment	Baseline	340	Week 2	350		
Control	No treatment	Baseline	370	Week 2	380		
Stressed	No treatment	Baseline	315	Week 2	320		
Stressed	No treatment	Baseline	375	Week 2	375		
Stressed	No treatment	Baseline	380	Week 2	380		
Stressed	No treatment	Baseline	315	Week 2	315		
Stressed	No treatment	Baseline	370	Week 2	370		
Stressed	No treatment	Baseline	305	Week 2	310		
Stressed	Saline	Baseline	330	Week 2	340	Week 7	355
Stressed	Saline	Baseline	335	Week 2	335	Week 7	355
Stressed	Saline	Baseline	325	Week 2	320	Week 7	345
Stressed	Saline	Baseline	315	Week 2	315	Week 7	330
Stressed	Saline	Baseline	325	Week 2	325	Week 7	340
Stressed	Saline	Baseline	345	Week 2	350	Week 7	365
Control	Venlafaxine	Baseline	355	Week 2	370	Week 7	395
Control	Venlafaxine	Baseline	325	Week 2	345	Week 7	365
Control	Venlafaxine	Baseline	320	Week 2	330	Week 7	345
Control	Venlafaxine	Baseline	330	Week 2	345	Week 7	360
Control	Venlafaxine	Baseline	360	Week 2	380	Week 7	390
Control	Venlafaxine	Baseline	320	Week 2	330	Week 7	350
Stressed	Venlafaxine	Baseline	330	Week 2	330	Week 7	345

Stressed	Venlafaxine	Baseline	320	Week 2	325	Week 7	325
Stressed	Venlafaxine	Baseline	340	Week 2	340	Week 7	360
Stressed	Venlafaxine	Baseline	300	Week 2	300	Week 7	310
Stressed	Venlafaxine	Baseline	340	Week 2	345	Week 7	365
Stressed	Venlafaxine	Baseline	335	Week 2	345	Week 7	365
